# Multiple molecular logic gate arrays in one system of (2-(2′-pyridyl)imidazole)Ru(ii) complexes and trimeric cyclophanes in water†‡

**DOI:** 10.1039/d2sc03617g

**Published:** 2022-08-26

**Authors:** Chao-Yi Yao, Hong-Yu Lin, Philip Morgenfurt, Tia E. Keyes, A. Prasanna de Silva

**Affiliations:** School of Chemistry and Chemical Engineering, Queen's University Belfast BT9 5AG UK a.desilva@qub.ac.uk; School of Chemical Sciences, Dublin City University Dublin 9 Ireland

## Abstract

Shape-switchable cyclophane hosts allow the controlled capture and release of reactive polypyridineRu(ii) complexes in water. This gives rise to a network of host–guest binding, acid–base reactions in ground and excited states, and chemical redox interconversions. In the case of (2-(2′-pyridyl)imidazole)Ru(ii) complexes, several molecular logic gate arrays of varying complexity emerge as a result. Cyclophane-induced ‘off–on’ switching of luminescence in neutral solution is found to originate from two features of these aromatic hosts: enhancement of radiative decay by the polarizable host and the suppression of nonradiative decay involving deprotonation by reducing the water content within the deep host cavity. These are examples of nanometric coordination chemistry/physics being controlled by inclusion in an open box. The aromatic units of the macrocycle are also responsible for the shape-switching mechanism of wall collapse/erection.

## Abstract 2

Transactions of a polypyridineRu(ii) complex with photons, protons and shape-switchable hosts show several types of molecular logic.

## Graphical abstract

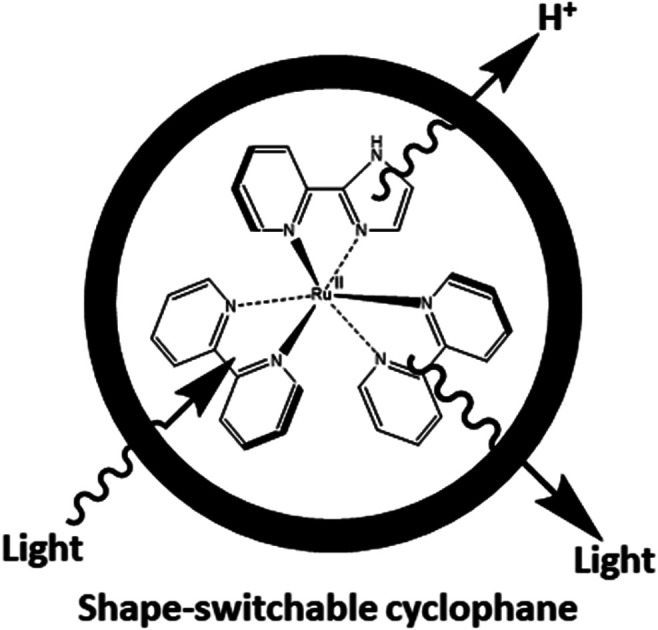

## Introduction

Luminescent switching/sensing/logic research has been driven more by atomic rather than by molecular inputs,^1–6^ in terms of numbers of publications, although notable exceptions concerning sugars, proteins and oligonucleotides do exist.^7–26^ This situation has arisen because of the dearth of suitable receptors/hosts for molecular targets *cf.* those for atomic counterparts.^27^ In the case of polypyridineRu(ii) lumophores,^28–30^ those responding to H^+^,^31–41^ Na^+^,^41–43^ Cl^−^,^44,45^ Ni^2+^,^46–48^ and even electrons^49,50^ outnumber those switching with glucose,^51^ H_2_PO_4_^−^,^52^ MoO_4_^−^.^53^ Cases involving O_2_-induced quenching^54,55^ have not been included in this group because no definable receptor exists. Generally, polypyridineRu(ii) lumophores need to be outfitted with a receptor (directly or *via* a spacer) or a pyridine moiety needs to be mutated into another heterocycle in order to confer the property of luminescence switchability. On the other hand, the recent appearance of large macrocyclic hosts which inclusively capture polypyridineRu(ii) complexes^56–58^ promises a supramolecular approach to luminescent switching, although host-induced ‘off–on’ switching has been noted only once in a summarized form.^58^ However, luminescent ‘off–on’ switching of an Ir(iii) complex is known.^20^ Such sharp binary switching is useful for molecular logic and computation.^59–74^ A hydrogen-bonded capsule capable of including polypyridineRu(ii) complexes^75^ unfortunately cannot operate in water.

PolypyridineRu(ii) complexes which are studied here contain one or more 2-(2′-pyridyl)imidazole ligands so that their optical properties become pH-dependent *via* the ionizable imidazole N–H bond.^31,33,39,40^ So, a second avenue of H^+^-induced ‘off–on’ switching of luminescence opens up. Such switching has a long history.^76,77^ Another theme of this work is the switching of binding induced by redox or by protonation/deprotonation.^78–80^ Although many such individual instances are known,^66^ here we present a unique occasion where all four of these switching types converge (Table 1) so that a set of molecular logic gate arrays are produced. We exploit the fact that molecular guests have more channels for stimulation once they are bound to hosts, as compared to atomic guests.

**Table 1:** The different types of ‘off–on’ switches examined

Input	Output
Host (1–5)	Guest (6) luminescence
H^+^	Guest (6) luminescence
Redox	Host (3/4)–guest (6) binding
H^+^	Host (1 and 2)–guest (7) binding

Nanometric coordination chemistry, *e.g.* deprotonation equilibria and kinetics, and its physics, *e.g.* radiative rates, of these large metal complexes are now significantly controlled for the first time by putting them in an open box or by denying them access when the box is shut. This is achieved by combining time-resolved and steady-state luminescence studies on coordination complex 6 as the nanometric object with/without hosts 1 and 2. Remarkably, the ‘box’ is a macrocycle rather than a molecular cage, where the aromatic walls of the macrocycle demarcate a substantial 3-dimensional space from where water is excluded.

## Results and discussion

**Fig. 1:**
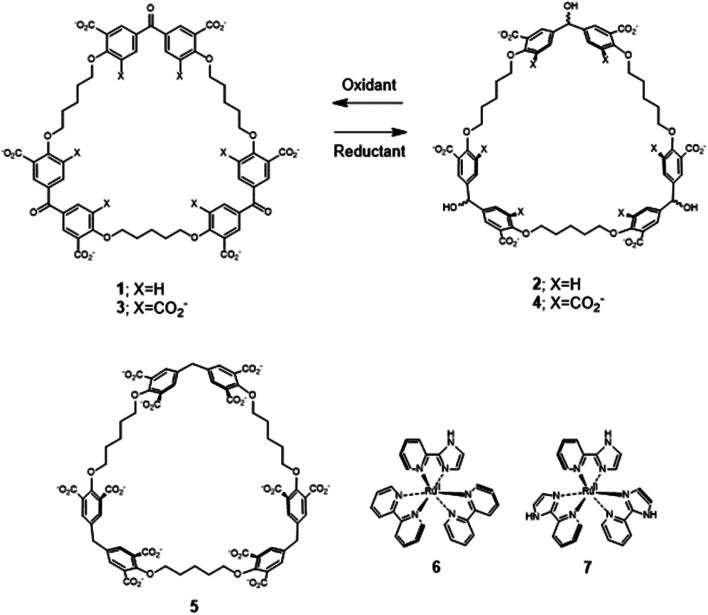
Molecular structures of the hosts 1–5 and guests 6 and 7. No stereochemistry is intended.

Recently, we described the trimeric cyclophanes 2 and 4 (Fig. 1) and their ability to selectively include (bipyridine)_3_Ru(ii) and (phenanthroline)_3_Ru(ii) complexes in water, as opposed to small cationic and neutral aromatics, with submicromolar affinities in some cases.^58^ The redox partners of trialcohols 2 and 4 – the triketones 1 and 3 respectively – were also studied, as was a control host 5 (Section S1‡). These cyclophanes consist of three pairs of phenylenes straddling a ‘corner’ composed of a secondary alcohol, a ketone or a methylene. There are also three pentamethylene linkers with ether oxygen termini which complete the large macrocycle. Inclusive binding was found with hosts 2, 4 and 5 whereas perching binding was seen with host 1. Following the initial work,^58^ it was natural to examine polypyridineRu(ii) complexes carrying an ionizable group as the first mutation of the parent guests. The imidazole N–H is an example because of its increased acidity owing to electron withdrawal by Ru(ii), which is augmented in the MLCT (metal to ligand charge transfer) excited state.^33^ Guest 6 is a good place to start because its luminescence and other properties have been well-established.^31,39,40^ A preliminary summary of some of our findings concerning 6 without/with hosts 1–4 was mentioned in ref. 58. Guest 6 and host 5 have not been combined previously, except in passing. To carry the mutation of the parent guest compounds further, 7 (ref. 81 and 82) is also studied now but with cyclophanes 1 and 2 only. Here, the number of ionizable groups is increased from one to three. As synthesized, 7 exists as a mixture of *mer* and *fac* isomers in a 3 : 1 ratio.^82^

### ^1^H NMR spectroscopy

**Fig. 2:**
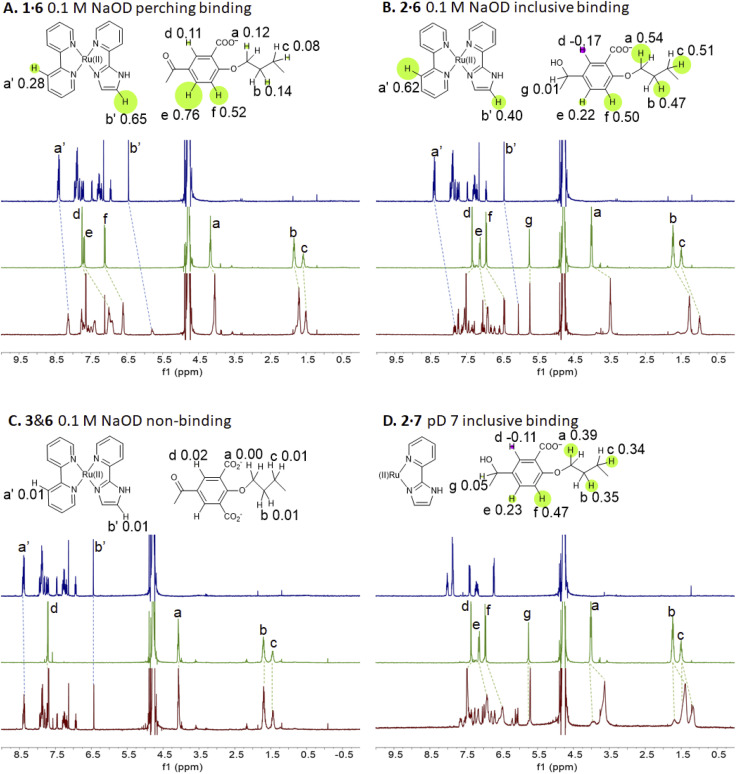
(A–D) ^1^H NMR spectra of guest (blue), host (green) and their combination (red) at 10^−3^ M in 0.1 M NaOD/D_2_O at 27 °C, except that panel D is at pD 7. All binding-induced chemical shift changes are indicated. −Δ*δ* values are given on partial molecular structures and their relative magnitudes are illustrated by the radii of circles centred on each proton. Negative or positive Δ*δ* values are shown by green or red circles respectively. Δ*δ* maps indicate binding modes, *e.g.* inclusive, perching or non-binding. The molecular structures indicate only one of the bipyridines and the heteroligand for illustrative purposes.

Representative spectra of guests 6 and 7 and trimeric cyclophanes 1–5 alone and in binary mixtures are shown in Fig. 2. The complexation-induced chemical shift differences (Δ*δ* values) are given in the insets as Δ*δ* maps on the partial molecular structures. The conveniently assignable protons are marked. It is clear that trialcohols 2 and 4 and control host 5 induce significant upfield shifts on all the protons of guest 6, indicating that inclusive or nesting binding has occurred in neutral water. Also, the upfield shifts induced in the pentamethylene linker protons of all three hosts are due to paramagnetic shielding caused by the bipyridyl units of guest 6 facing them. As would be expected in an inclusive complex, there are small downfield shifts induced in the corner protons and in the 3-phenylene protons (Fig. S1‡). The orientation of guest 6 within 2 is such that each polypyridine ligand edge is held at a benzhydrol corner of the host.^58^ Although all samples have been annealed for 1 hour at 60 °C, the spectra of the mixtures display some broadening at 27 °C.

In passing, we note that in the presence of guest 6, the more anionic trialcohol 4 shows a component (50%) which is not exchanging with the 6-bound form on the NMR timescale (Fig. S1‡). This is not surprising because a macrocyclic dodecacarboxylate will have some un-ionized carboxylic acid groups at pD 7. Linear polyacrylates display this behaviour^83^ and a macrocyclic version is expected to have an even stronger effect owing to the electric fields being unable to dissipate by chain extension. A reasonable deduction is that this causes intra-annular hydrogen bonding between CO_2_H and CO_2_^−^ groups so that the cavity is unavailable for rapidly including 6. Furthermore, such protons are held in a water-poor region in the host cavity so that their exchange with a CO_2_^−^ group in a copy of 4 already engaged with guest 6 would be hindered. This was also observed when host 4 engaged with (bipyridine)_3_Ru(ii).^58^ Host 2, with only half the number of potential CO_2_^−^ groups of 2, does not show such an effect in this case. This analysis would be strengthened if host p*K*_a_ values and 2D NMR spectra were available but these are too complex to resolve.

**Fig. 3:**
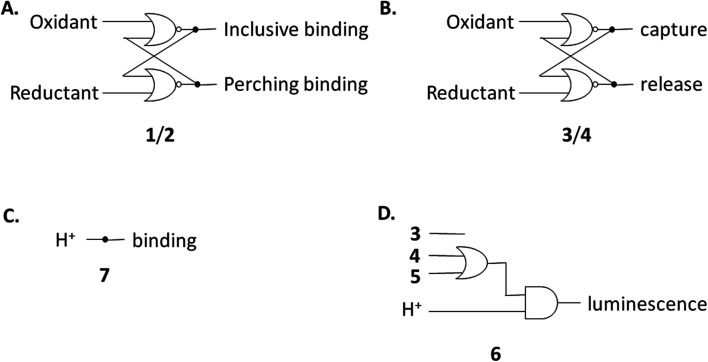
(A–D) Various logic gate arrays emerging from this study.

The redox partner of trialcohol 2 is triketone 1. It also induces upfield shifts in all the protons of guest 6. However, the pattern of effects induced by guest 6 on host 1 is diametrically opposite to what was induced on host 2. Now, large upfield shifts are induced in all the phenylene protons of host 1 because of paramagnetic shielding caused by the bipyridyl units of guest 6 facing them. The pentamethylene protons of host 1 only experience tiny Δ*δ* values, suggesting that the edges of bipyridyl units of guest 6 are pointed at them. The line broadening mentioned above is also largely absent. Unlike the inclusive binding of 6 within trialcohol 2, we now have perching binding of 6 on triketone 1. The terms ‘perching’ and ‘nesting’ were introduced by Cram by analogy with a bird on a limb.^84^ Such switching of binding mode between two host redox partners 1 and 2 was seen with (bipyridine)_3_Ru(ii) and (phenanthroline)_3_Ru(ii) previously.^58^ This arises because trialcohols have larger cavities with phenylene walls standing orthogonal to the mean macrocycle plane. On the other hand, the triketones force pi-conjugation with the phenylenes (limited by cross-conjugation) so that at least one phenylene will come into the mean macrocycle plane along with each carbonyl group.^85^ Perching binding is known in previous cases where the macrocycle cavity is constricted, although the individual interactions are noticeably different.^86–90^ Since we have previously shown that redox interconversions of alcohols and ketones correspond in logic terms to a Reset-Set flip-flop,^85^ the two different modes of inclusive binding with trialcohol 2 and perching binding with triketone 1 can be represented as outputs in this situation (Fig. 3A). Briefly, RS flip-flop behaviour means that each ‘high’ input drives an individual output to be ‘high’. A fresh application of the same ‘high’ input makes no difference to the outputs. A given set of outputs are maintained when neither input is ‘high’. Importantly, both inputs are not allowed to be ‘high’ simultaneously.

Triketone 3, the redox partner of trialcohol 4, shows no evidence of binding with guest 6 in Δ*δ* values. So, ‘off–on’ switching of binding by redox stimulus is demonstrated here since the fully ionized component of trialcohol 4 bound guest 6 successfully. This binary action can also be shown as a RS flip-flop (Fig. 3B) where the outputs are capturing and releasing actions corresponding to trialcohol 4 and triketone 3 respectively. Why does the hexacarboxylate triketone 1 manage perching binding whereas dodecacarboxylate triketone 3 essentially fails to bind 6? The presence of six extra CO_2_^−^ groups in host 3 minimizes hydrophobic patches in the phenylenes so that pi–pi/pi–C–H interactions with pyridine units of guest 6 become untenable. However, the hydrophobic equatorial belt is preserved in trialcohols 2 and 4 and control host 5 because the hydrophobic effect is an important component of the binding interaction between these hosts and guest 6.

The situations described above don't change much when the spectra of guest 6 in the presence of hosts 1 or 2 are examined in 0.1 M NaOD (Fig. S1‡), although free 6 is expected to deprotonate since it's p*K*_a_ value is 7.9.^31^ Nevertheless, the p*K*_a_ value of cyclophane-bound 6 would likely be significantly higher, if p*K*_a_ perturbations in anionic micelle microenvironments are anything to go by.^91–93^ Indeed, these values can be as high as 10.6 (Table 2) and will be discussed in later sections. Nevertheless, it appears that the deprotonated form of 6 is tolerated as a guest by hexacarboxylate hosts 1 and 2. We note some complications seen in the NMR spectra of 6 with dodecacarboxylate hosts 4 and 5 in Table 2, footnote *k*, which suggests that the tolerance begins to wear off for more anionic hosts. In these two instances, Δ*δ* values are noticeably smaller.

**Table 2:** Optical and binding properties of 6 without/with cyclophanes 1–5 in H_2_O or D_2_O

Property	6	1·6	2·6	3 and 6	4·6	5·6
*λ*_Abs_(acid)/nm	463	465	465	465	467	467
*ε*(acid)/10^3^ M^−1^ cm^−1^	10.0	10.0	11.0	11.0	10.0	10.0
*λ*_Abs_(base)/nm	486	492	493	486	486	486
*ε*(base)/10^3^ M^−1^ cm^−1^	9.4	9.6	10.0	10.0	9.7	10.0
*λ*_Isos_/nm	474	480	480	475	477	478
*λ*_Lum_(acid)/nm	638	631	633	631	630	631
10^2^*ϕ*(acid)^a^	1.1	1.0^h^	1.3^h^	1.5	2.1	1.6
*λ*_Lum_(base)/nm^b^	688	686	687	685	687	686
10^2^*ϕ*(base)	0.1	0.2	0.2	0.1	0.1	0.1
log *β*_NMR_^c^	—	4.6	5.6	<2^i^	4.8	4.8
log *β*_NMR_^d^	—	4.6	5.5	<2^i^	k	k
log *β*_Lum_^e^	—	4.5	5.5	<2^i^^,^^j^	4.6	4.9
LE_Cyclophane_^f^	—	11	13	j	18	20
p*K*_a Abs_	8.8	9.1	9.6	9.0	10.4	10.6
p*K*_a Lum_	5.5, 8.8	h, 9.0	h, 9.5	5.5, 9.0	*ca.* 6, 10.1	j, 10.5
LE_H+_^g^	7.2, 2.0	h, 4.7	h, 5.8	5.2, 4.3	1.05, 22	j, 21

a Luminescence quantum yield.

b Uncertainty ±3 nm.

c pD 7.0, D_2_O, 27 °C.

d 0.1 M NaOD, D_2_O, 27 °C.

e At pH 7.0, H_2_O, room temperature, the corresponding log *β*_Lum_ value in 0.1 M NaOH is immeasurable due to insignificant change in the property.

f At pH 7.

g Ratio of total intensity from plateau to plateau.

h Limited by precipitation of 1 and 2 at pH < 6.5.

i Immeasurably small due to insignificant change in the property within the concentration range studied.

j Undetectable or immeasurable due to the absence of a significant luminescence enhancement step.

k In the case of 4·6, analysis of Δ*δ* values for a fraction of the aliphatic protons gives log *β*_NMR_ = 5.8 (the other fraction having Δ*δ* = 0), but all the aromatic protons of host and guest give insignificant induced shifts. This suggests non-inclusive binding under these conditions. Similarly, 5·6 gives log *β*_NMR_ = 4.4.

Now we shift attention to potential guest 7 which contains three imidazole units. The NMR Δ*δ* values are substantial at pD 7 (Fig. 2D) but they are negligible in 0.1 M NaOD (Fig. S1‡), because the multi-anionic form of 7 obtained by ionization of some of the three imidazole N–H bonds would be repelled by multi-anionic hosts.^94^ So, this is ‘off–on’ switching of binding of 7 by pH. In logic terms,^59–74^ this corresponds to a YES operation where the input is H^+^ and the output is host–guest binding (Fig. 3C). Many examples of this general type are known, *e.g.* ammonium ions are bound to crown ethers whereas the corresponding Bronsted bases, the amines, are not.^95–97^

### UV-visible absorption spectroscopy

**Fig. 4:**
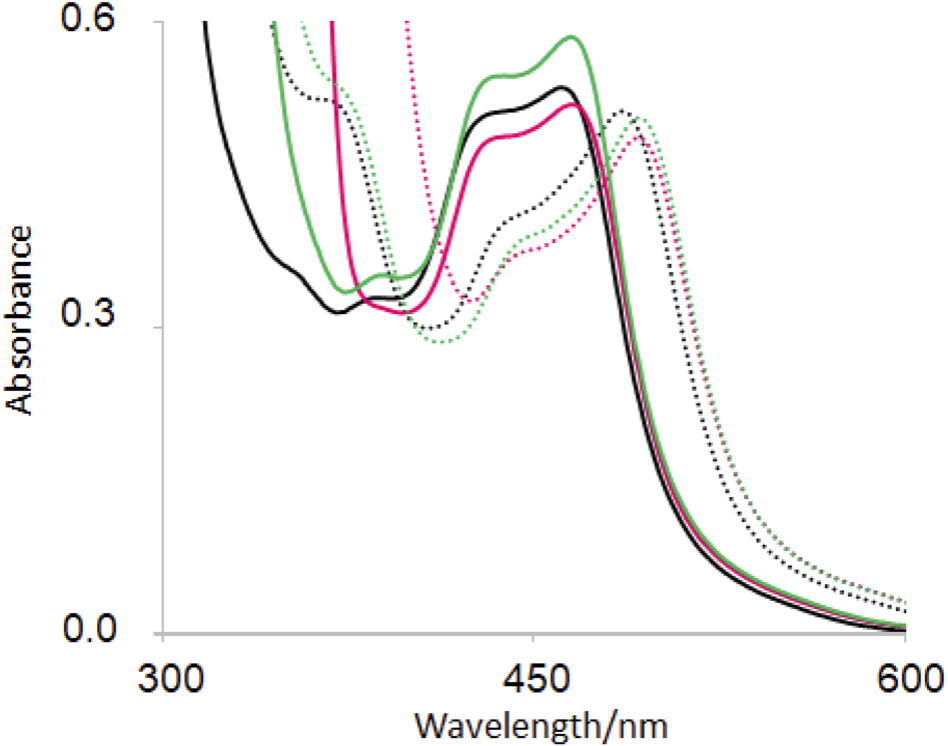
UV-visible absorption spectra of 6(black), 2·6 (green) and 1 6 (red) in H_2_O at pH 7 (full line) and at pH 12 (dotted line).

The base-induced red-shift of 23 nm of guest 6 (ref. 31) (Table 2) has been noticed before. In basic solution, the MLCT excited state of 6 involving charge transfer from Ru(ii) to the bipyridines can be stabilized by pi-donation from the electron-rich pyridylimidazolate ligand. Representative spectra under neutral and basic conditions without/with hosts are shown in Fig. 4.

In recent work, hosts 1–5 were found to have negligible influence on the MLCT absorption band of (bipyridine)_3_Ru(ii).^58^ Perhaps this wasn't surprising, since UV-visible spectra of polypyridineRu(ii) complexes are not strongly affected by polarity of the medium. Then it is also not surprising that the *λ*_Abs_(acid) value of guest 6 is only slightly red-shifted with hosts (Table 2). In contrast, *λ*_Abs_(base) value is red-shifted by 6–7 nm for hexacarboxylate hosts 1 and 3 only. From the pH-dependent NMR spectroscopic studies discussed above, we know that host–guest binding survives in basic solution for these cases just as they do under neutral conditions. The pyridylimidazolate ligand presents an anion at the edge of a cavity already lined with six carboxylates, which should lead to significant destabilization of the ground state. Upon excitation of deprotonated 6, electron density from the N^−^ is delocalized into other parts of the guest so that the excited state suffers less of the aforementioned destabilization. So, the host-induced red-shift originates from the hexanionic nature of hosts 1 and 2. Inclusive binding of 6 within trialcohol 2 is expected to provide such an environment, but how does perching binding of 6 on triketone 1 fit such an outcome? Δ*δ* values from NMR spectroscopy provides the answer. In the case of inclusive complex 2·6, a substantial paramagnetic shift (Δ*δ* = −0.40) is seen for the proton on the carbon adjacent to deprotonated N–H (marked as b′ in Fig. 2). Although triketone 1 holds 6 in a perching complex with some exposure to water, the b′ proton displays a larger paramagnetic shift (Δ*δ* = −0.65). So the imidazolate N^−^ is held very close to the face of the host phenylenes with their carboxylate appendages. The host-induced red-shift is naturally smaller when we consider the pyridylimidazole ligand in neutral solution (Fig. 4).

Fig. 4 also shows a host-induced increase in extinction for 2·6 in neutral medium, indicating a change to the dielectric environment of 6 due to inclusion within host 2. Perching complex 1·6 is not efficient in this regard. This effect disappears even for 2·6 In basic medium, suggesting an altered conformation although it remains as an inclusive complex according to NMR evidence (Table 2).

### Steady-state luminescence spectroscopy

Hosts 1–5 provide a less polar environment for guest 6 and so it is gratifying to see that the emission [*λ*_Lum_(acid)] is blue-shifted by 7 nm in all five cases (Table 2). However, the *λ*_Lum_(base) values are unaffected. The different host-induced behaviours in absorption and emission wavelengths in both the acid and base forms of guest 6 deserve comment, because related effects are known in fluorophores due to other supramolecular interactions like solvation^1,98,99^ and metal-binding.^1,100^ Internal charge transfer (ICT) excited states of fluorophores can be weakly affected by polar solvents regarding absorption wavelengths whereas large red shifts appear in emission. This is because solvent reorganization to stabilize the dipolar excited state occurs during the excited state lifetime.^1^ In passing, we note that when ‘fluorophore-receptor’ systems develop a positive pole during excitation, embedding a metal ion causes an electrostatic repulsion and hence a blue-shift manifests in the absorption spectrum. However, the metal ion suffers a ‘reorganization’ during the excited state lifetime and moves out of the receptor.^101^ Thus the emission spectrum shows no metal-induced blue-shift.^100^

The acid form of 6 involves a degree of pi-donation from imidazole to stabilize the MLCT state involving Ru(ii) and the bipyridines. So we have a positive pole developing on imidazole and a negative pole on the bipyridines. In the host-free situation, this will require rotational relaxation of water for stabilization during the lifetime of the excited state. Such solvent relaxation would be minimal when guest 6 is held by the hosts due to exclusion of water. That is why a blue-shift relative to the host-free case is found for all hosts 1–5. Even the MLCT state of (bipyridine)_3_Ru(ii) is stabilized by water in a rather similar way and so, host-induced blue-shifts are seen here too.^58^ The base form of 6 has a weaker emission which leads to a larger uncertainty of the *λ*_Lum_(base) value (Table 2) so that its host-induced effects cannot be evaluated.

It is important to note that the static charge repulsions which caused host-induced red-shifts observed in absorption are overwhelmed by the rotational relaxation of water dipoles which occurs over picosecond timescales and so would be complete after the lifetime of MLCT excited states of 6. Such interplay of dipolar (or dielectric) and electric field effects are well-known in micelle microenvironments,^91–93^ though their different timescales are less discussed.

**Fig. 5:**
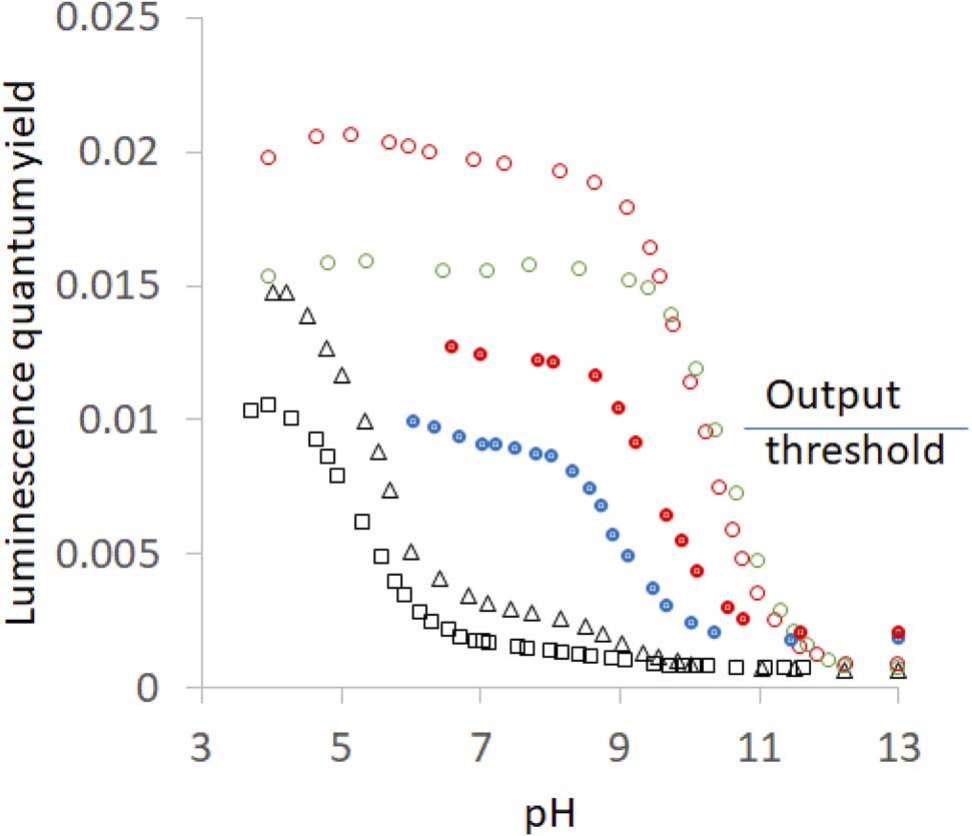
Luminescence quantum yield (*φ*)-pH profiles in aerated H_2_O for 6 without any host (black open squares), with host 2(red filled circles), with host 4 (red open circles), with host 1 (blue filled circles), with host 3 (black open triangles) and with host 5 (green open circles). The threshold for the luminescence output at pH 7 between ‘high’ and ‘low’ states is shown. Some of the profiles are from ref. 58.

Hosts 1–5 also enhance the luminescence intensity of polypyridineRu(ii) complexes by offering a degree of shielding of their excited states from water.^102^ Luminescence enhancement (LE) factors of up to 3.3 were found for *e.g.* (bipyridine)_3_Ru(ii).^58^ As Fig. 5 and Table 2 show, the LE values are much larger for 6 at pH 7. Indeed, such order-of-magnitude enhancements can be regarded as ‘off–on’ light switches.^58,103^ This represents a four-input logic gate array (Fig. 3D) where inputs 4 and 5 supply an OR gate which feeds an AND gate (whose other input is H^+^) to generate the luminescence output. In other words, this is enabled OR logic^63^ where H^+^ is the enabling input. Input 3 has no effect. This molecular logic gate array is unprecedented, to the best of our knowledge. We note that hosts 1 and 2 are not included in this analysis since their LE values are moderate. In the absence of hosts, 6 deprotonates while still in the excited state and the luminescence switches ‘off’ when the pH exceeds *ca.* 5.5.^31,39,40^ Presence of a host pushes this pH value much higher. This will be discussed below after quantitation of the effects.

In passing, we note that switching ‘on’ of luminescence of 6 by cyclophanes is rather reminiscent of the ‘off–on’ enhancement of phosphorescence of 2-bromonaphthalene by β-cyclodextrin under certain conditions.^104–107^ The switching ‘on’ of f–f luminescence of Eu^3+^ or Tb^3+^ by polyazamacrocycles^108,109^ is also related. All these cases share an inclusion of the emitter despite phenomenological and mechanistic disparities.

### Luminescence lifetime determinations

As observed previously,^31,39,40^6 has a very short excited state lifetime owing to its N–H deprotonation from the MLCT excited state (Section S4,‡Table 3). Our novel finding is that host 1, and even more so host 2, lengthens the major component of the lifetimes by a large factor, which is larger at pH 7 than at pH 12 (Fig. S5‡). So, N–H deprotonation of 6 is suppressed by perching binding to host 1 and by inclusive binding to host 2. (Bipyridine)_3_Ru(ii) shows smaller host-induced enhancement factors for its luminescence lifetime, which are pH-independent. The host-free value is in line with previous measurements.^110,111^ These results parallel the findings of host-induced enhancement factors for luminescence intensities discussed in previous sections and for quantum yields collected in Table 2.

**Table 3:** Luminescence lifetimes (*τ*, ns, with amplitude percentages in brackets), luminescence quantum yields (*ϕ*) and related kinetic parameters (*k*_r_ and *k*_nr_, 10^6^ s^−1^) of 6 and (bipyridine)_3_Ru(ii) without/with cyclophanes 1 and 2 in aerated H_2_O at pH 7 and 12^a^

Property	Species	pH 7	pH 12
*τ*	6	29(94%), 331(6%)	30(96%), 388(4%)
*τ*	1·6	115(81%), 309(19%)	62(94%), 454(6%)
*τ*	2·6	147(91%), 386(9%)	60(95%), 520(5%)
*τ*	(Bipyridine)_3_Ru(ii)	406	408
*τ*	1· (Bipyridine)_3_Ru(ii)	733	724
*τ*	2· (Bipyridine)_3_Ru(ii)	748	744
10^2^*ϕ*	6	0.17	0.08
10^2^*ϕ*	1·6	0.94	0.21
10^2^*ϕ*	2·6	1.2	0.21
10^2^*ϕ*	(Bipyridine)_3_Ru(ii)	4.2	4.1
10^2^*ϕ*	1· (Bipyridine)_3_Ru(ii)	9.7	9.7
10^2^*ϕ*	2· (Bipyridine)_3_Ru(ii)	11.8	11.9
*k*_r_, *k*_nr_	6	0.059, 30	0.025, 30
*k*_r_, *k*_nr_	1·6	0.082, 7.2	0.034, 15
*k*_r_, *k*_nr_	2·6	0.082, 5.6	0.035, 15
*k*_r_, *k*_nr_	(Bipyridine)_3_Ru(ii)	0.10, 1.6	0.10, 1.6
*k*_r_, *k*_nr_	1·(Bipyridine)_3_Ru(ii)	0.13, 0.9	0.13, 0.9
*k*_r_, *k*_nr_	2·(Bipyridine)_3_Ru(ii)	0.16, 0.7	0.16, 0.7

a *k*_r_ and *k*_nr_ values are calculated from the major component with the shorter lifetime under each set of conditions and the corresponding luminescence quantum yield according to the equations given in Section S4.

The luminescence decay of 6 contains a minor (4–19%) component which is longer-lived (300–500 ns) and whose contribution decreases with increasing pH. This represents the undeprotonated form of 6. As shown in previous sections, hosts 1 and 2 bind 6 in both its deprotonated and undeprotonated forms.

Radiative (*k*_r_) and nonradiative (*k*_nr_) rate constants can be extracted from these data (Table 3). For 6 in neutral water, hosts cause rather large suppressions of *k*_nr_ associated with N–H deprotonation. Smaller, but still significant, host-induced enhancements (×1.4) of *k*_r_ are also found. This is due to the immediate environment of 6 being changed from water to the more polarizable pi-system of the hosts. Indeed, the Strickler–Berg expression for *k*_r_ is proportional to the square of the refractive index,^112^ whose ratio for anisole and water is 1.3.^113^

A major conclusion is that the visually dramatic ‘off–on’ switching of 6 in neutral solution^58^ is caused by host-induced effects on *k*_nr_ and on *k*_r_ operating in tandem. The effect on *k*_nr_ is moderated in alkaline solution because of the N^−^ coupling strongly to the few available water molecules whereas N–H would not do so.

In the case of (bipyridine)_3_Ru(ii), host-induced effects on *k*_r_ are similar to those found for 6, but are pH-independent. Both aspects are expected from the discussion in previous sections. The effects on *k*_nr_ are smaller (×2) than those seen for 6, but these are still significant and pH-independent. The contrast between the data for 6 and (bipyridine)_3_Ru(ii) shows the greater rigidity imposed by host inclusion is not responsible for the host-induced suppression of *k*_nr_.

### Equilibrium constant determinations

The host–guest binding interactions seen above can be quantitated *via* NMR spectroscopy by investigating the dependence of Δ*δ* values on concentration, followed by analysis according to eqn (1).^114^

1 (Δ*δ*/Δ*δ*_max_)/[1 − (Δ*δ*/Δ*δ*_max_)]^2^ = *βa*

where ‘*a*’ is the concentration of prospective host, for a 1 : 1 stoichiometry. This stoichiometry has been proven for these hosts and polypyridineRu(ii) complexes.^58^ Cyclophanes and guests are maintained at 1 : 1 molar ratios. The binding constants (*β*) obtained are collected in Table 2. ‘Off–on’ switching of binding of 6 by redox stimulus upon the host system 3/4 is quantitatively illustrated by the *β* values differing by at least 3 orders of magnitude. The contribution of hydrophobicity towards binding of 6 is demonstrated by the higher *β* value seen for the more hydrophobic host 2, *cf.*4.

These binding interactions can also be quantitated *via* luminescence spectroscopy by investigating the dependence of intensities on host concentration, followed by analysis according to eqn (2).^114,115^

2 [(*I*_L_ − *I*_Lmin_)/(*I*_Lmax_ − *I*_L_)] = *β*{*a* − *b*[(*I*_L_ − *I*_Lmin_)/(*I*_Lmax_ − *I*_Lmin_)]}

where ‘*a*’ is the concentration of host and ‘*b*’ is the concentration of guest for a 1 : 1 stoichiometry.

Proton-binding is also an important component of the present work. This was investigated *via* pH-dependent absorbance (*A*) in UV-visible absorption spectra, analyzed according to eqn (3).^114,116^

3 log[(*A* − *A*_min_)/(*A*_max_ − *A*)] = pH − p*K*_a_

where *K*_a_ is the acid dissociation constant of the imidazole N–H bond.

pH-dependent luminescence intensities (*I*_L_) or quantum yields (*ϕ*) can also be analysed with a version of eqn (3), where the absorbance is replaced by the necessary luminescence variable.

log *β* values can be determined by NMR spectroscopy in neutral and alkaline water, whereas the luminescence method is only successful under neutral conditions. Deprotonated 6 has a luminescent quantum yield (*ϕ*) which is smaller by an order-of-magnitude than the acid form (Table 2), due to coupling of the imidazolate N^−^ with water molecules to open a vibrational loss pathway which has been demonstrated by studies in D_2_O.^39^ Such pathways are known for organic fluorophores^117^ and for lanthanide ions.^118^ It is gratifying that both methods return near-identical values for log *β*. It is also interesting that log *β* values are essentially constant in neutral and alkaline conditions for each host–guest pair. Inclusive binding (2·6) turns out to be *ca.* 10 times stronger than perching complexation (1·6) for the more hydrophobic hosts. The log *β* value for 2·6 corresponds to a nearly micromolar affinity, although (bipyridine)_3_Ru(ii) binds 2 with a 10-fold higher *β* value.^58^ The less hydrophobic hosts 4 and 5 inclusively bind guest 6 about 10-fold weaker, confirming the contribution of hydrophobicity to the host–guest interaction in water. However, the corresponding triketone 3's log *β* value is estimated as <2. This quantitates the redox-induced switching ‘off–on’ of binding of 6 as being worth at least 3 log units.

The ground state p*K*_a_ values of 6 with/without hosts, determined *via* absorbance from Fig. 4 and relatives, can be understood in terms of dielectric and electrostatic effects exerted by the hosts. This approach^119^ has a history of success when applied to micelle environments.^91–93,120,121^ Accordingly, a low dielectric environment compared to water would discourage an ionization of the N–H group. Also, local negative charges would amplify the local concentration of protons which would also discourage an ionization of the N–H group. Thus, both effects would act in unison to raise the p*K*_a_ value of 6. Greater the exposure of 6 to water, the smaller this p*K*_a_ increase would be.

Host-free 6 has a p*K*_a_ value of 8.8 under our conditions, and the presence of almost non-binding 3 gives a value (9.0) which is within experimental error (±0.1). The value produced by the perching complex 1·6 (9.1) is not much higher, because of significant exposure of the guest and its N–H group to water. Inclusive complexation of guest 6 within the more hydrophobic host 2 would produce a large dielectric effect but the presence of only six carboxylates would produce a relatively small electrostatic effect. In practice, the Δp*K*_a_ value is 0.8. Inclusive complexes 4·6 and 5·6 involving the less hydrophobic hosts 4 and 5 would produce a smaller dielectric effect but the presence of twelve carboxylates would produce a relatively large electrostatic effect. Hence, Δp*K*_a_ values of 1.6 and 1.8 respectively, are found. Host 5 lacks the OH groups at the corners and thus has a slightly larger dielectric effect than host 4 does. So it has the largest Δp*K*_a_ value of all. In other words, the p*K*_a_ series for guest 6 is: 5> 4> 2> 1> 3 = free.

The p*K*_a_ values measured by luminescence almost exactly follow those determined by absorbance, *i.e.* ground state p*K*_a_ values are found in all cases, although their contributions differ. However, luminescence by its very nature, can uncover excited state p*K*_a_ values if the ionization occurs adiabatically. Fig. 5 shows inflection points corresponding to this situation in some cases. The precipitation of the more hydrophobic hosts prevents a full examination of the acidic pH range, but important observations and deductions can still be made. Host-free 6 is free of this encumbrance and the inflection point is found at 5.5.^40^ This says that the deprotonation of the N–H group occurs in the excited state. As might be expected, the same inflection is found in the presence of the non-binding 3. Careful examination of the region around pH 6 in Fig. 5 reveals a barely perceptible step for host 5 and a clear, but very small, step for host 4. It can also be estimated from the plateau heights in Fig. 5 that any contribution from a step around pH 5.5 would have increasingly larger contributions in the cases of hosts 2 and 1, despite precipitation preventing measurements. In other words, the series for guest 6 is: 5< 4< 2< 1< 3 = free. Remarkably, this is the opposite of the order seen in the previous paragraph and confirms the role of the same blend of dielectric and electrostatic effects in switching the deprotonation pathway as well. This is the first time that the same structure–activity relationship is found for host-induced changes in a guest's p*K*_a_ values and for host-induced partitioning of excited- and ground-state deprotonations.

When 6 is fully exposed to water, the deprotonation occurs in the excited state because water molecules and buffer anions can support the charge separation by dielectric relaxation. As the hosts push out water or as the hosts tie up water molecules by local electric fields arising from the CO_2_^−^ groups, there is less support for the charge separation during N–H ionization. So the ionization is late and occurs after deexcitation of the excited state back to the ground state. The H^+^-induced switching ‘on’ of 6's emission at pH 7 in the presence of a host can be understood as arising from host-induced displacements of emission-pH profiles along the pH axis. We note, in passing, that such displacements and other perturbations of emission-pH profiles correspond to several logic types. H^+^, Ca^2+^,^122^ H^+^, lipase,^123,124^ and H^+^, Na^+^-driven cases^125,126^ are available.

Since the acidity of 6 can be influenced by excitation, like other coordination complexes^33^ and many other organic compounds,^127–129^ it is natural to consider a thermodynamic cycle to relate excited- and ground-state acidities. Application of such Förster cycles to our case requires several assumptions^127^ which are not always easy to justify, but an estimate^31^ is given here for comparison with experiment.4
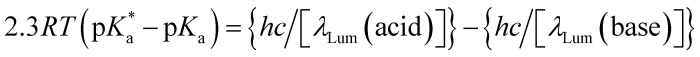
where *h* is Planck's constant and *c* is the velocity of light. p*K*_a_ and 
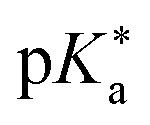
 are the fully equilibriated values for the ground and excited states respectively. For host-free 6, this gives 
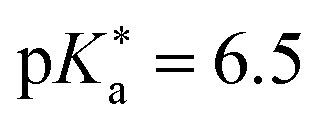
 by substituting appropriate values from Table 2 into eqn (4). The experimental pH at the inflection point is 5.5. The calculation of the experimental 
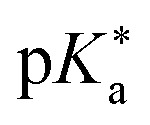
 value requires luminescence lifetimes (*τ*) according to eqn (5),^127^ which are available in a previous section (Table 3). This produces a 
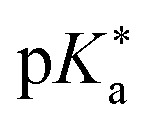
 value of 6.6, in good agreement with the value obtained from the Förster cycle. This is to be compared with a literature value of 5.6 (ref. 40) referred to different conditions.5
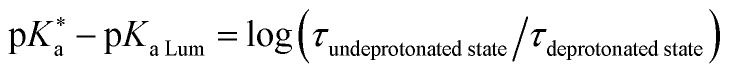


However, the acidity of 6 can also be influenced by complexation by the cyclophane hosts. This allows a different and less common thermodynamic cycle^130^ to be considered. Analysis of the scheme in Fig. S4‡ according to a thermodynamic cycle gives eqn (6).

6 p*K*_a bound_ − p*K*_a free_ = log *β*_NH_ − log *β*_N^−^_

where *K*_a bound_ and *K*_a free_ refer to the acid dissociation constants of 6 with and without hosts respectively. *β*_NH_ and *β*_N^−^_ refer to the binding constants of the hosts and 6 in its acid or base form respectively.

In the present work, the data in Table 2 suggests that eqn (6) is not well-obeyed. For example, the p*K*_a_ value of 6 changes from 8.8 to 9.6 upon binding host 2. Then the left hand side of eqn (6) becomes 0.8. However, the log *β* values for 2·6 in neutral and alkaline solution differ only by 0.1. This discrepancy is possibly because entropy terms are not well represented in such thermodynamic cycles of the Hess' law type.^131^

**Fig. 6:**
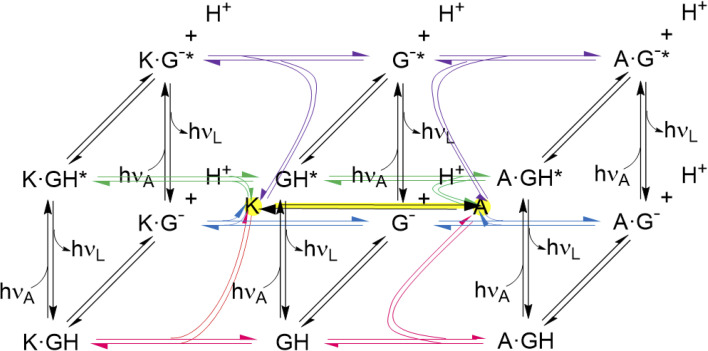
Network of host–guest complexation–decomplexation equilibria, guest deprotonation–protonation equilibria, guest excitation–deexcitation and *ex situ* host redox interconversion. The latter is shown with a yellow highlight. The free hosts are shown with yellow highlights at the center of each cube. Further study should reveal conditions for *in situ* host redox interconversion, as has been achieved for these hosts and guests like tris(bipyridine)Ru(ii),^58^ when the network would grow extra connections between nodes. The three black vertical panels are Förster cycles^127–129^ for the guest alone and when complexed with the triketone host (K) and trialcohol host (A). The bottom panels are cases of Fig. S4.‡ The guest and its deprotonated form are GH and G^−^ respectively. Excitation is indicated with *. The photons *hν*_A_ and *hν*_L_ are for absorption and luminescence respectively and are characteristic for each species. Host protonation–deprotonation is not considered, so as to limit the network size, although some relevant results are described in the text. Host excitation–deexcitation is outside the scope of this paper, although dimeric versions of hosts 1 and 2 have been studied previously in this way.^65^

The thermodynamic cycles corresponding to eqn (4) and (6) are parts of a network of reactions unearthed by the present work (Fig. 6). These reactions include host–guest binding, acid–base reactions in the ground and excited states, as well as redox-induced host interconversions. It is a pleasure to note that remarkably complex networks have been constructed from photo-, pH-, thermal- and host-responsive flayylium salts.^132,133^ The behaviour of some of these also correspond to various logic gate arrays. Peptide reaction networks are similarly complex and amenable to incorporation within logic schemes as well.^134^

## Conclusions

The kinetics and equilibria of a polypyridineRu(ii) complex's unimolecular reactivity are shown to be controlled by binding with shape-switchable hosts for the first time. At short timescales, multianionic hosts 1–5 exert electrostatic effects on guest reactions/processes, which show up as red-shifts in absorption spectra. At longer times, hosts 1–5 suppress strong dielectric effects due to water on guest reactions/processes. Thus, blue-shifts are found in emission spectra and a change from excited state deprotonation to ground state deprotonation is seen, leading to an ‘off–on’ light switch in neutral water. In particular, system (1–5)·6 forms an unprecedented reaction network composed of guest excitation–deexcitation, guest deprotonation–protonation, host–guest binding-unbinding, as well as redox interconversion of hosts. Several of the stimulus-response patterns correspond to molecular logic gate arrays of different types.

## Data availability

The data that support the findings of this study are available in the ESI.‡

## Author contributions

C. Y. Y. synthesized 1, 2, 6 and 7. H. Y. L. synthesized 3, 4 and 5. C. Y. Y. performed all the studies and data analyses on 1–7, except for luminescence lifetime determinations and resonance Raman spectroscopy which were conducted and analysed by P. M. under the supervision of T. E. K., A. P. de S. conceived the project, analysed the data and wrote the paper, with assistance from C. Y. Y.

## Conflicts of interest

There are no conflicts to declare.

## Supplementary Material

SC-013-D2SC03617G-s001
