# Supplementary material for: Multiple molecular logic gate arrays in one system of (2-(2′-pyridyl)imidazole)Ru(ii) complexes and trimeric cyclophanes in water
Source: Chem Sci. 2022 Aug 26;13(36):10856–67. doi: 10.1039/d2sc03617g (PMC9491216; doi:10.1039/d2sc03617g)
Supplement: SC-013-D2SC03617G-s001 [file SC-013-D2SC03617G-s001.pdf]

Electronic Supplementary Material (ESI) for Chemical Science.
This journal is © The Royal Society of Chemistry 2022

Electronic Supplementary Information

Multiple Molecular Logic Gate Arrays in One System of (2-(2'-pyridyl)imidazole)Ru(II) Complexes and Trimeric Cyclophanes in Water

Chao-Yi Yao, Hong-Yu Lin, Philip Morgenfurt, Tia E. Keyes and A. Prasanna de Silva

Materials and methods

Section S1. Syntheses

Hosts **1**-**5**[58] and guests **6**[31]&**7**[82] are prepared and characterized as given in the literature. Full synthetic procedures are given within these references. The dichloride salt of (bipyridine)$_3$Ru(II) is obtained commercially.

Section S2. ^{1}H NMR spectroscopy

Bruker DRX 400 and DRX 600 spectrometers are used. Spectra are given in Figure S1, some of which are given here after adaptation from the electronic supporting information of ref. 58 for convenience of the reader. 10^{-3} M host and 10^{-3} M guest are employed.

Section S3. Steady-state luminescence spectroscopy

A Perkin-Elmer LS55 luminescence spectrometer is used. The influence of pH on 10^{-6} M **6** in the presence of 10^{-3} M **5** is shown in Figure S2. The influence of host **5** on 10^{-6} M **6** at pH 7 is shown in Figure S3.

The base-induced red-shift of 23 nm seen in the absorption spectrum of host-free **6** is stretched to 50 nm in the emission spectrum (Figure S2), as might be expected by the water reorganization during the excited state lifetime. The host-bound cases also show similarly large red-shifts.

Section S4. Luminescence lifetime determinations

Measurements are performed on $5x10^{-5}$ M Ru(II) complexes and 10^{-3} M host solutions in water at pH 7 or at pH 12 on a PicoQuant FluoTime 100 Compact FLS TCSPC system using a 450 nm pulsed laser source generated from a PicoQuant PDL800-B box and an external Thurlby Thandar Instruments TGP110 10 MHz pulse generator to enable acquisition of long lifetime data. A band-pass filter (>520 nm) is used to exclude excitation light. Data is collected up to 40,000 counts and decay curves are analysed using PicoQuant Fluofit software and tail-fit statistical modelling. In all cases the minimum number of exponents are used for the fit and Chi-squared values are employed to assess the goodness-of-fit to the exponential decay along with visual inspection of the residuals.

Representative cases are given in Figure S5.

Radiative (k_r) and non-radiative (k_{nr}) are calculated from the luminescence lifetimes (τ) and the quantum yields (ϕ) after allowing for the effects of air on the luminescence of **6**[40] and (bipyridine)$_3$Ru(II) [109,110] according to the following equations. The corresponding values in the absence of air are τ_0 and ϕ_0.

$$\phi = k_r/[k_r + k_{nr} + k_{O2}(O_2)]$$
$$\tau = 1/[k_r + k_{nr} + k_{O2}(O_2)]$$
$$\phi_0 = k_r/(k_r + k_{nr})$$
$$\tau_0 = 1/(k_r + k_{nr})$$
$$k_r = \phi/\tau = \phi_0/\tau_0$$
$$\tau_0/\tau = \phi_0/\phi$$

$k_{nr} = (1/\tau_0) - k_r$

Section S5. Resonance Raman spectroscopy

Resonance Raman spectroscopy is carried out on a Horiba Labram HR confocal microscope, using a 10x objective with a 473 nm diode laser (5 µW) as resonant excitation source. Conventional Raman is carried out on a Horiba Jobin Yvon HR800 UV spectrometer using 785 nm laser excitation. A 600 lines/mm diffraction grating is employed, providing data at 0.5 cm^{-1} resolution. The x-axis is calibrated versus the Rayleigh line (0 nm) and the phonon mode from silicon wafer (520.7 cm^{-1}). Typically, an exposure time of 4 s and accumulation for 6 s are used for spectral recording and spectra are collected across a 200–2000 cm^{-1} spectral range.

Spectra for **6** under various conditions are given in Figure S6. A spectrum for $(bipyridine)_3Ru(II)$ is included for comparison.

When excited at 474 nm, the resonance concerns **6**'s MLCT state involving Ru(II) and the bipyridines so that the imidazole modes are not observed among the Raman bands. Therefore, the spectra (Figure S6) of **6** and $(bipyridine)_3Ru(II)$[S1,S2] are essentially identical. Deprotonation of the imidazole N-H has an indirect effect on the MLCT state which shows up in the electronic spectra (e.g. Figure 4) but only very weakly in the resonance Raman spectrum where ca. 2 cm^{-1} shifts to lower frequency are observed in the signature bipyridine modes (Figure S6). Since the host-induced effects in Figure 4 are smaller than pH effects, it is understandable that hosts **1**&**2** show no noticeable influence on the resonance Raman spectrum of **6** (Figure S6).

S1. A. Basu, H. Gafney and T. Strekas, *Inorg. Chem*. 1982, **21**, 2231.
S2. A. Baiardi, C. Latouche, J. Bloino and V. Barone, *Dalton Trans*. 2014, **43**, 17610.

Section S6. Time-resolved emission anisotropy measurements

Time-resolved emission anisotropy measurements are carried out on the PicoQuant Fluotime 100 Time-Correlated Single Photon Counting (TCSPC) system (excitation wavelength of 450 nm with a 510 nm narrow band pass dielectric filter) using a Thurlby Thandar Instruments TGP110 10MHz pulse generator with sheet polarisers to select V polarized excitation from the diode source. The G-factor is estimated by tail matching the VV and VH emission decays. Typical pulse rates of the excitation source are $1x10^5$ s^{-1} with typical pulse widths of 300 ps. 20,000 counts were collected for each anisotropy measurement. All measurements are performed in duplicate. Time resolved luminescence anisotropy measurements are analyzed using Picoquant Fluofit software to yield anisotropy (rotational correlation time, ϕ_{rot}) values using the mono-exponential model shown in (Equation S1).

$$r(t) = r_0.\exp(-\tau/\phi_{rot}) \qquad \text{------ (S1)}$$

where r(t) is the time-dependent anisotropy, r_0 is the limiting anisotropy, τ is the luminescent lifetime of the sample and ϕ_{rot} is the rotational correlation time.

Representative cases are given in Figure S7.

Following a suggestion from a Referee, time resolved luminescence anisotropy measurements were conducted to provide a novel approach to investigate the binding of Ru(II) complex **6** with cyclophane hosts **1**&**2**. A weak anisotropy decay is noticeable when **6** is bound by host **1** or by host **2** in water. This reflects anisotropy relaxation due to the host-guest assembly and its solvation shell, where each host adds 1100 Da when bound to **6**. It also suggests that **6** cannot rotate when assembled inside each host. However, as seen in Figure S7, the rotational time constant could not be measured as it was shorter than the temporal limit of our instrumentation (1 ns). The addition of 1100 Da would be expected to

cause rotational correlation times in the picosecond range, as found in fluorophores bound to cyclodextrins.[S3] Importantly, host-free **6** shows no anisotropy decay. For comparison, stronger anisotropy decays in the microsecond range are seen[S4] when polypyridineRu(II) complexes are bound to an integrin of molecular mass 240,000 Da.

S3. P. Sen, D. Roy, S. K. Mondal, K. Sahu, S. Ghosh, and K. Bhattacharyya, *J. Phys. Chem. A* 2005, **109**, 9716.
S4. K. Adamson, C. Dolan, N. Moran, R. J. Forster and T. E. Keyes, *Bioconjugate Chem*. 2014, **25**, 928.

Section S7. Further studies on 7

Section 7a. ^{1}H NMR spectroscopy of **7**

7 tends to precipitate in phosphate buffer. Although perdeuterated versions of some other buffers[94] are available, phosphate is simplest at serving our overall needs of a wide pD range, especially for studies with guest **6**. Precipitation of **7** is found, though to a lesser extent, even in the presence of host **1**. However, host **2** protects guest **7** against interaction with phosphate. Still, logβ values of 5.4 and ca. 4.6 can be inferred for host-guest complexes **2·7** and **1·7** respectively, at pD 7 (see Table S1). The NMR $\Delta\delta$ maps of the hosts can be analyzed (Figure 2), but the guest signals are not easy to unravel.

Section 7b. UV-visible absorption spectroscopy of **7**

A base-induced red-shift is found for **7**[82] (Table S1). The λ_{Abs} value in the UV-visible absorption spectrum of **7** is not changed upon adding **1** or **2** (Table S1), once samples are well-annealed in 0.1 M NaOH. Since pH variation on samples so annealed show time-dependent spectra, quantitation of pH dependence to obtain pK_a values is not attempted.

Section 7c. Steady-state luminescence spectroscopy of **7**

Compound **7** remains non-emissive even in the presence of hosts **1** or **2**. Host-binding does not alter the energy of the low-lying metal-centred (MC) excited state responsible for this non-emissivity.[81] Each replacement of a bipyridine with a pyridyl imidazole reduces the ligand field strength and three such replacements makes the MC state thermally-accessible at room temperature.

Table S1. Optical and binding properties of **7** in the presence of cyclophanes **1** and **2** in H_2O or D_2O.

Property	**7**	**1·7**	**2·7**
λ_{Abs} (0.1 M NaOH)/nm	451	451	448
ε (0.1 M NaOH)/ 10^3 $M^{-1}cm^{-1}$	8.2	8.3	6.6
Logβ_{NMR}^{a}	-	4.6	5.4
Logβ_{NMR}^{b}	-	<2^c	<2^c

a. pD 7.0, D_2O, 27 ^oC. b. 0.1 M NaOD, D_2O, 27 ^oC. c. Immeasurably small due to insignificant change in $\Delta\delta$ values within the concentration range studied.

Section S8. Schematic representations of Inclusive (or nesting) and perching binding

Inclusive (nesting) and perching binding modes are represented schematically in Figure S8. These modes are deduced from the ^{1}H NMR $\Delta\delta$ maps in this paper (Figures 2 and S1) and in ref. 58, as well as ROESY spectra in ref. 58.

A. 2·6 pD 7 inclusive binding

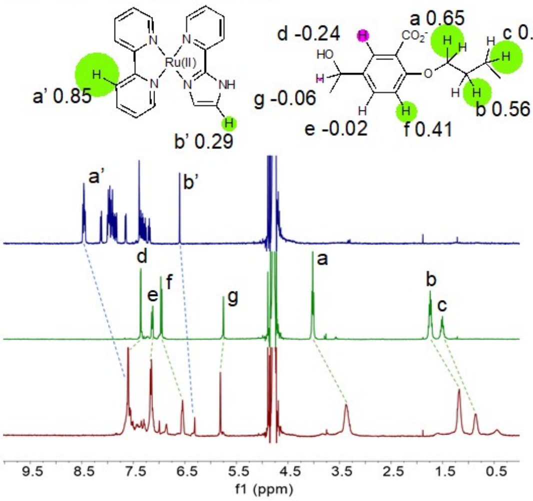

B. 4·6 pD 7 inclusive binding

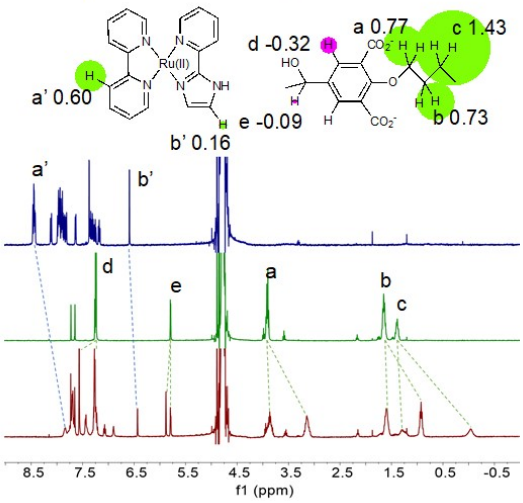

C. 4·6 0.1 M NaOD non-inclusive binding

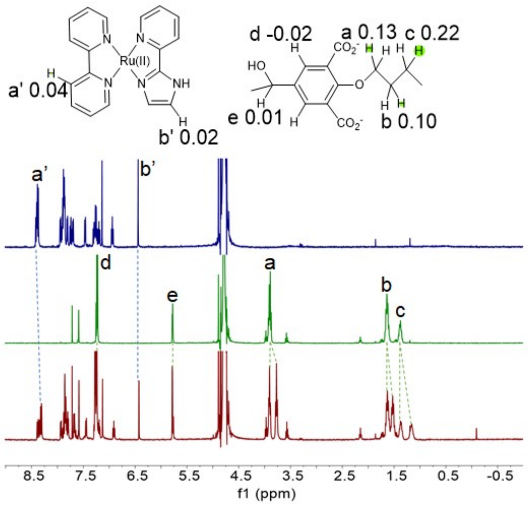

D. 1·6 pD 7 perching binding

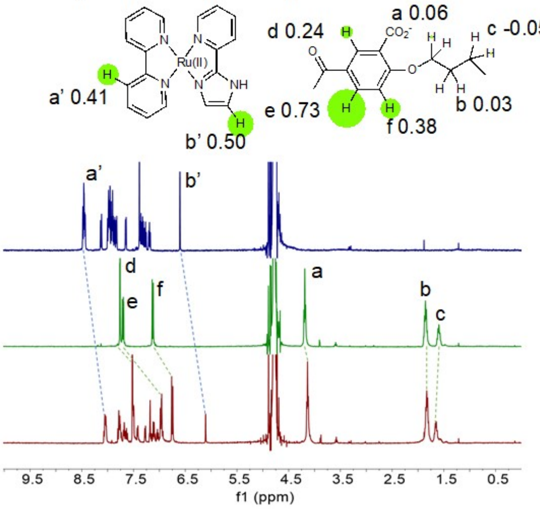

[continued]

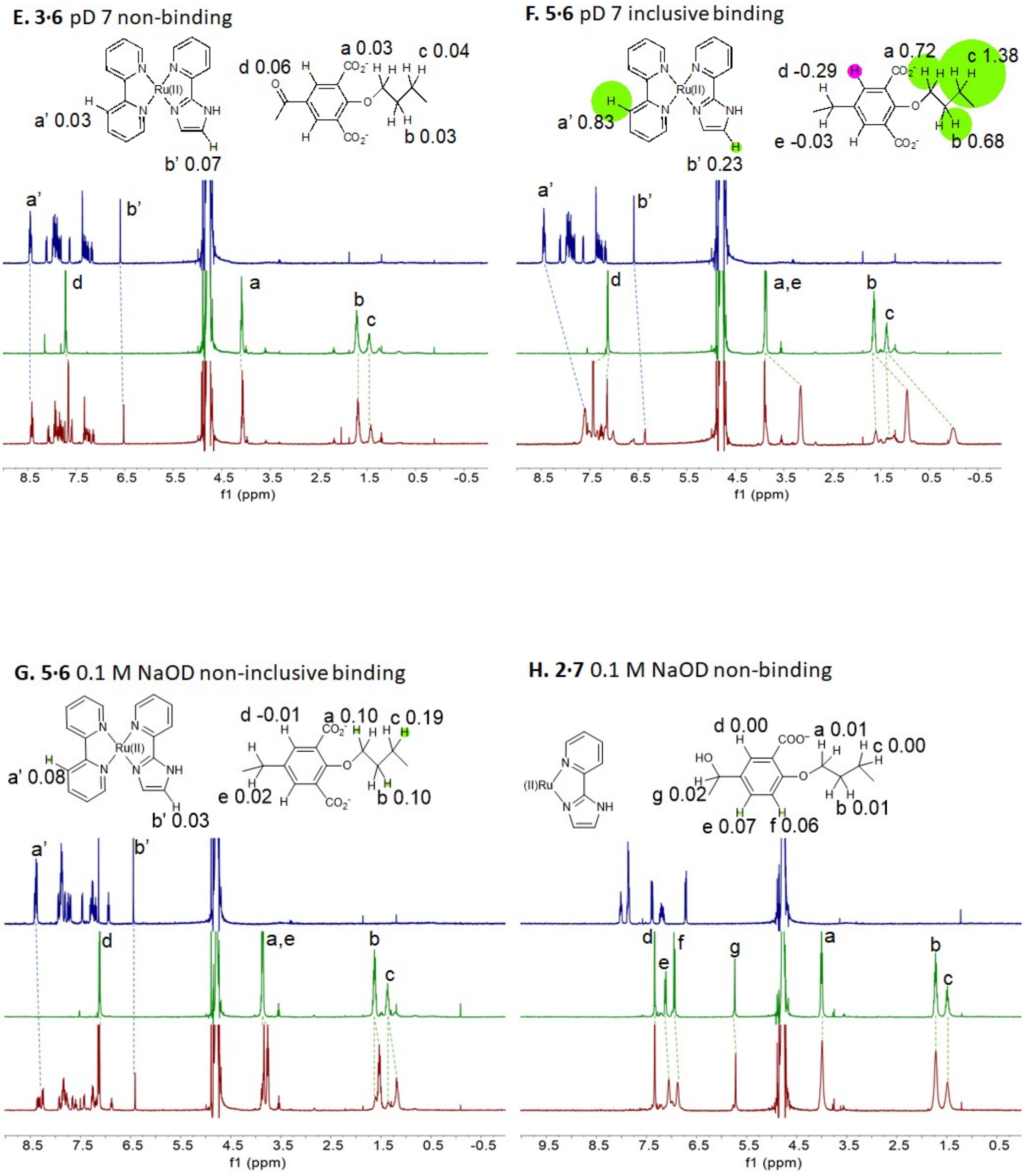

Figure S1. ^{1}H NMR spectra of guest (blue), host (green) and their combination (red). Other details are given in the caption to Figure 2.

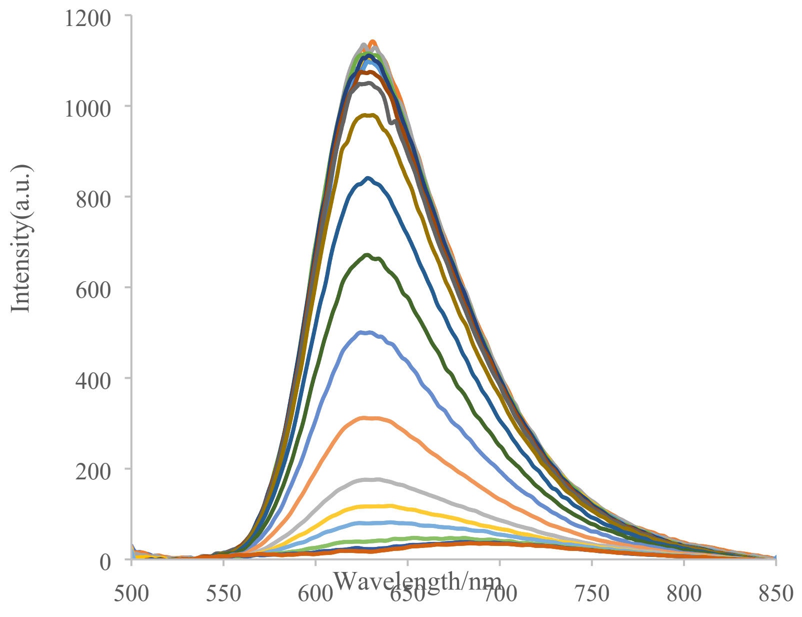

Figure S2. pH-dependent luminescence spectra of 10^{-6} M **6** in water in the presence of 10^{-3} M **5**. pH in order of decreasing intensity: 4.0. 4.8, 5.4, 6.5, 7.1, 7.7, 8.4, 9.1, 9.4, 9.7, 10.1, 10.4, 10.7, 11.0, 11.3, 11.5, 11.7, 12.0, 12.2 and 13.0.

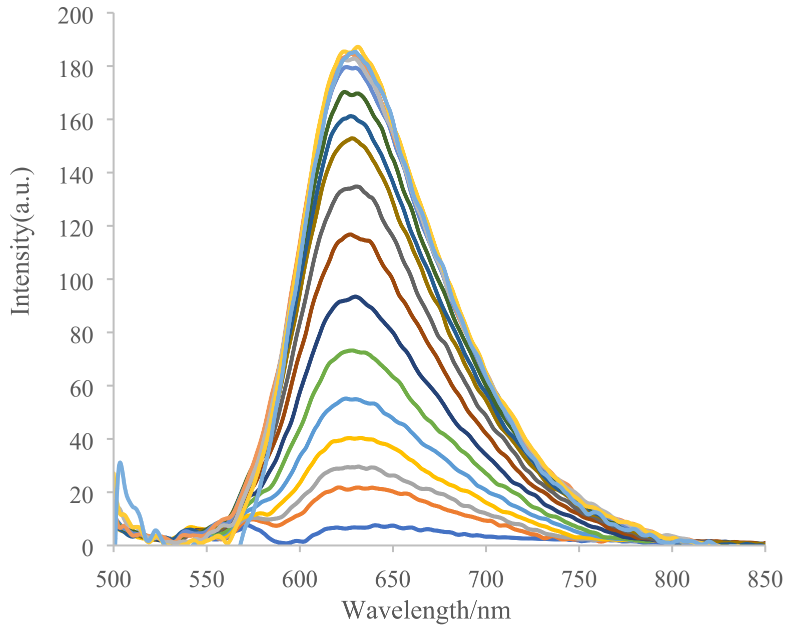

Figure S3. Host(**5**) -dependent luminescence spectra of 10^{-6} M **6** in water at pH 7. Host concentration in order of increasing intensity: 0, 1, 1.6, 2.5, 4, 6.3, 10, 16, 25, 40, 63, 100, 160, 250, 400, 630 and $1000x10^{-6}$ M.

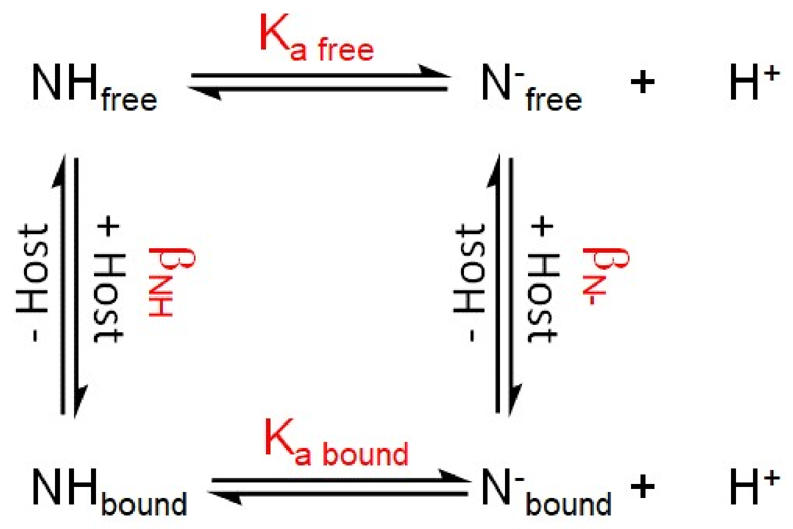

Figure S4. Thermodynamic cycle for intersecting host-guest and deprotonation-protonation equilibria.

Main Plot

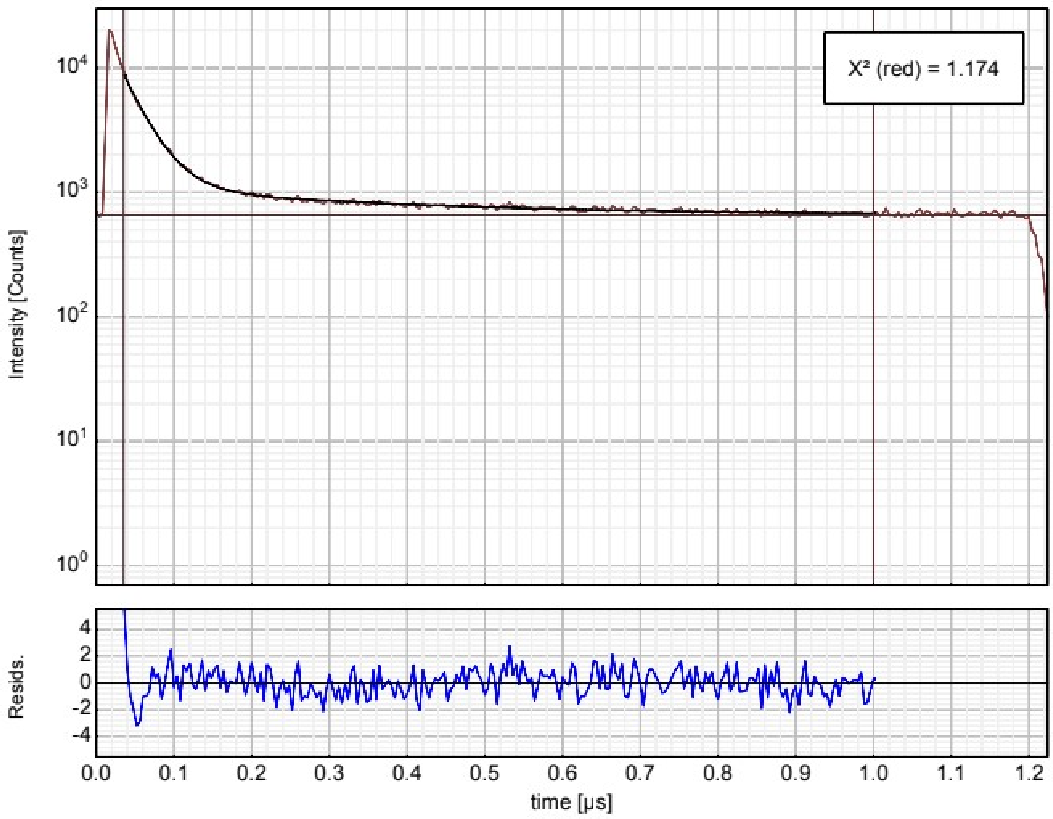

$$I(t) = \sum_{i=1}^{n} A_i \, e^{-\frac{t}{\tau_i}}$$

Parameter	Value	Conf. Lower	Conf. Upper	Conf. Estimation
A_1 [Cnts]	7601.4	-96.3	+96.3	Fitting
τ_1 [μs]	0.029717	-0.000391	+0.000391	Fitting
A_2 [Cnts]	450.2	-15.0	+15.0	Fitting
τ_2 [μs]	0.3213	-0.0123	+0.0123	Fitting
Bkgr. $_{Dec}$ [Cnts]	656.51	-4.88	+4.88	Fitting

Figure S5. A. Luminescence decay for5x10^{-5} M **6** in water without host at pH 7.

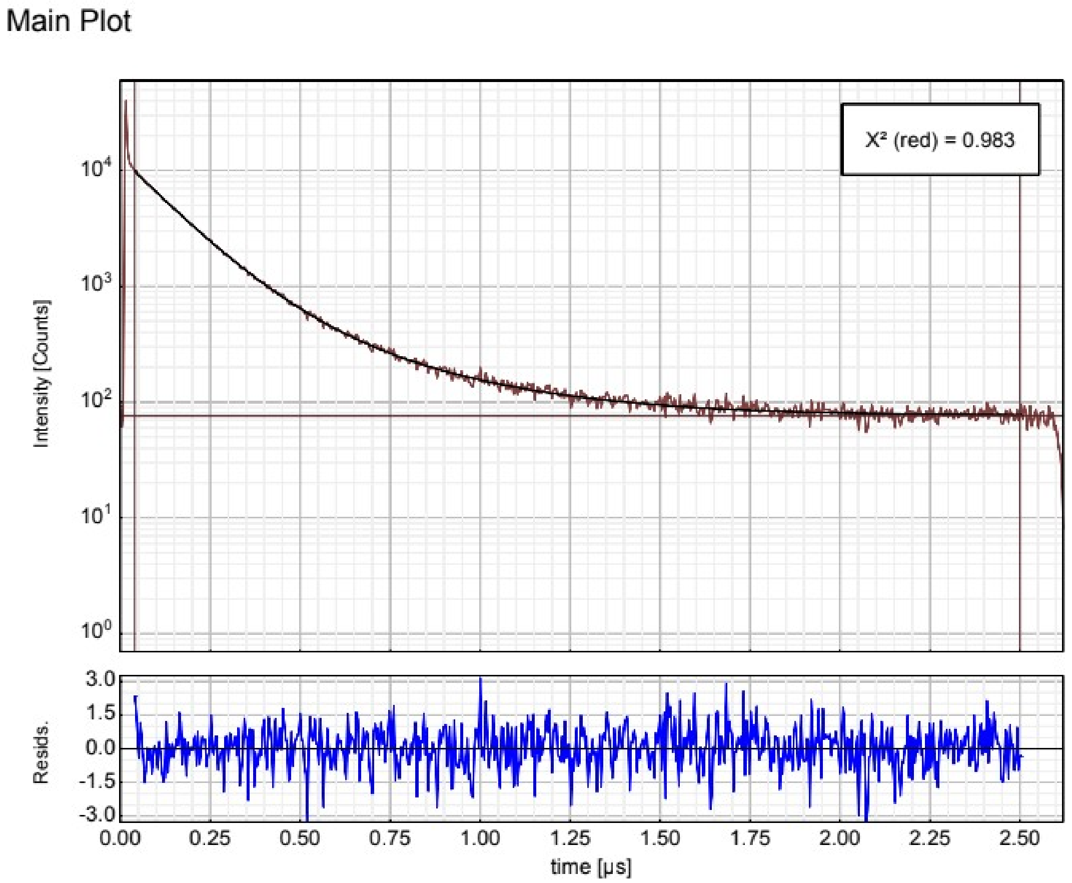

$$I(t) = \sum_{i=1}^{n} A_i \, e^{-\frac{t}{\tau_i}}$$

Parameter	Value	Conf. Lower	Conf. Upper	Conf. Estimation
A_1 [Cnts]	1040.5	-24.4	+24.4	Fitting
τ_1 [µs]	0.36116	-0.00552	+0.00552	Fitting
A_2 [Cnts]	8744.5	-70.4	+70.4	Fitting
τ_2 [µs]	0.132941	-0.000938	+0.000938	Fitting
Bkgr. Dec [Cnts]	75.88	-1.81	+1.81	Fitting

Figure S5. B. Luminescence decay for 5x10^{-5} M **6** in water with 10^{-3} M host **1** at pH 7.

Main Plot

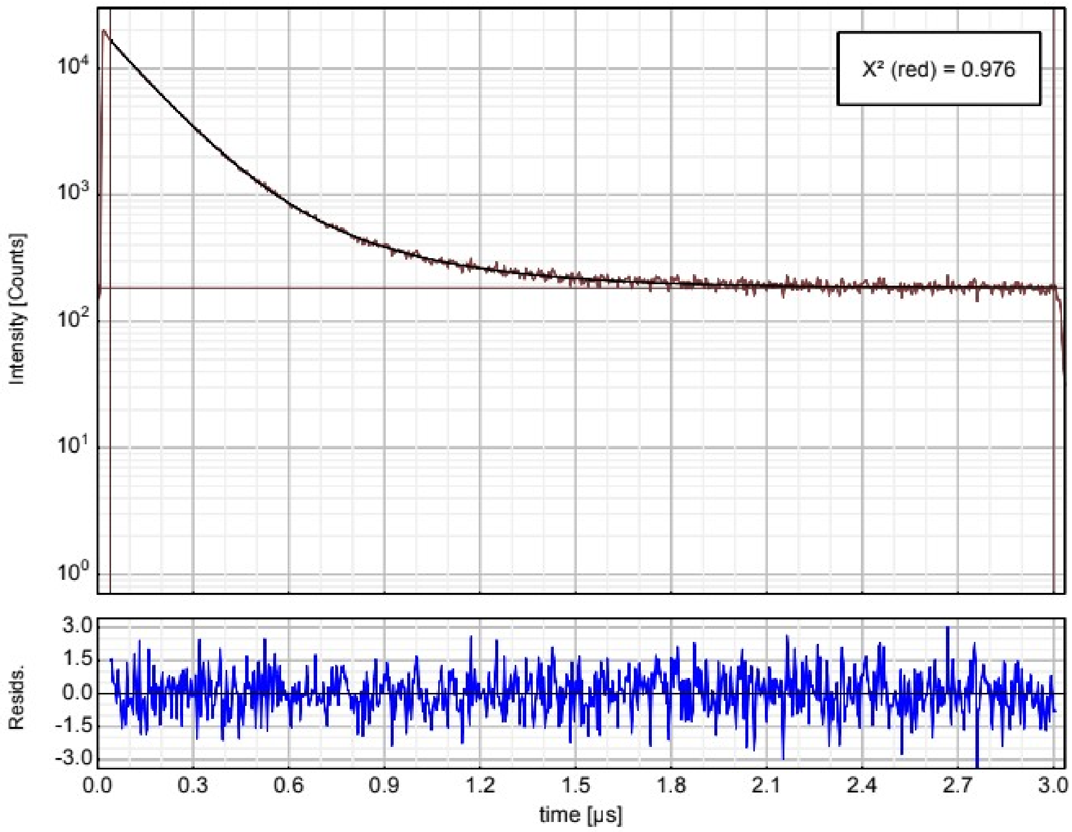

$$I(t) = \sum_{i=1}^{n} A_i \, e^{-\frac{t}{\tau_i}}$$

Parameter	Value	Conf. Lower	Conf. Upper	Conf. Estimation
A_1 [Cnts]	1520.5	-34.5	+34.5	Fitting
τ_1 [μs]	0.38355	-0.00588	+0.00588	Fitting
A_2 [Cnts]	14898.3	-95.0	+95.0	Fitting
τ_2 [μs]	0.146204	-0.000813	+0.000813	Fitting
Bkgr. $_{Dec}$ [Cnts]	183.97	-2.59	+2.59	Fitting

Figure S5. C. Luminescence decay for $5x10^{-5}$ M **6** in water with 10^{-3} M host **2** at pH 7.

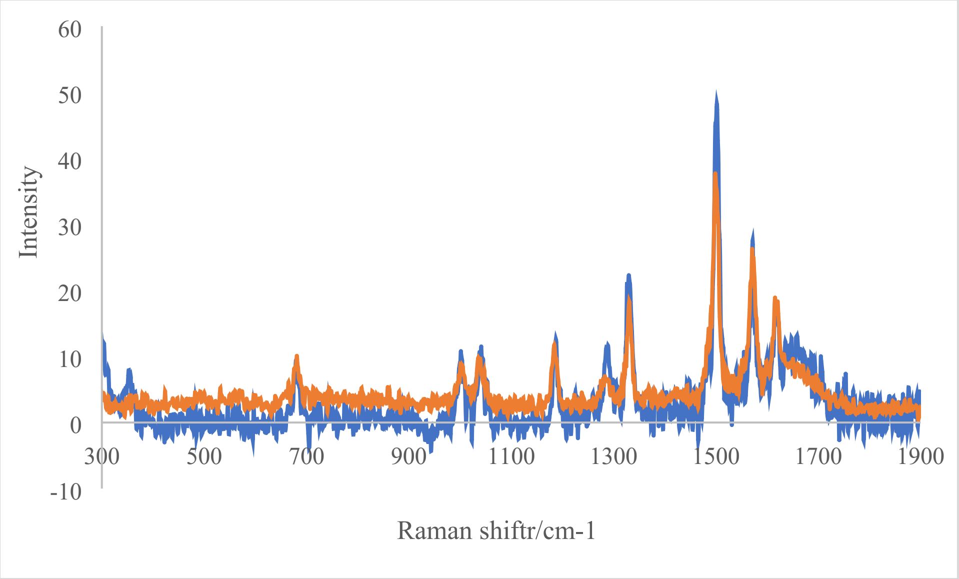

Figure S6. A. Resonance Raman spectra of 5x10^{-5} M **6** (orange) and 5x10^{-5} M (bipyridine)$_3$Ru(II) (blue) in water at pH 12.

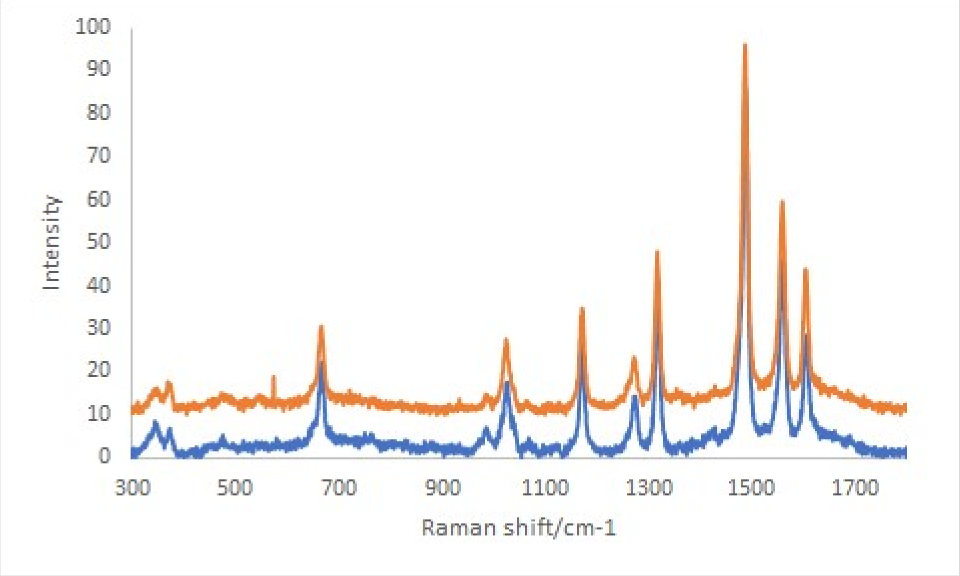

[continued]

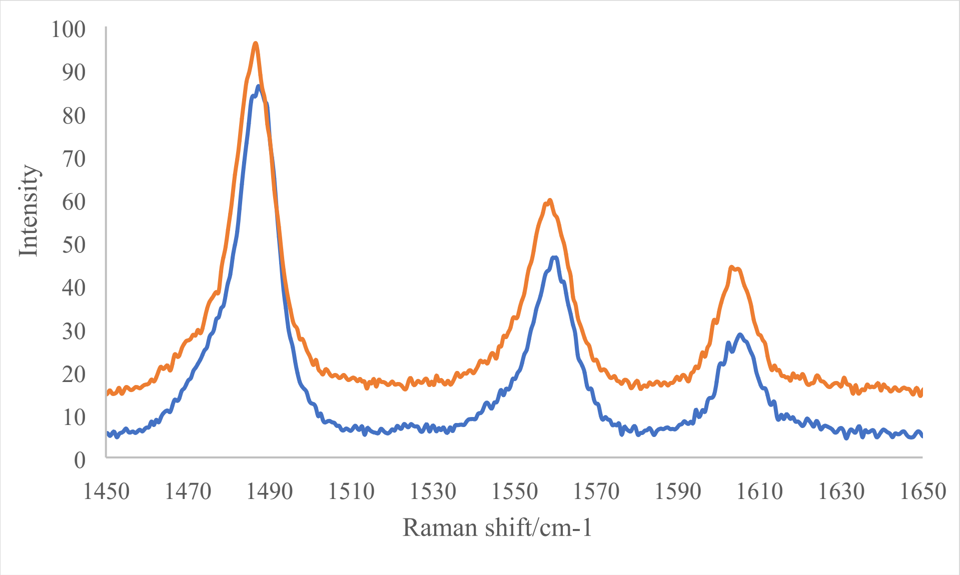

Figure S6. B. Resonance Raman spectra of 10^{-3} M **6** in water at pH 12 (orange) and at pH 7 (blue). It is easier to see the small, but noticeable, movement to lower frequencies at pH 12 in the zoomed-in lower panel.

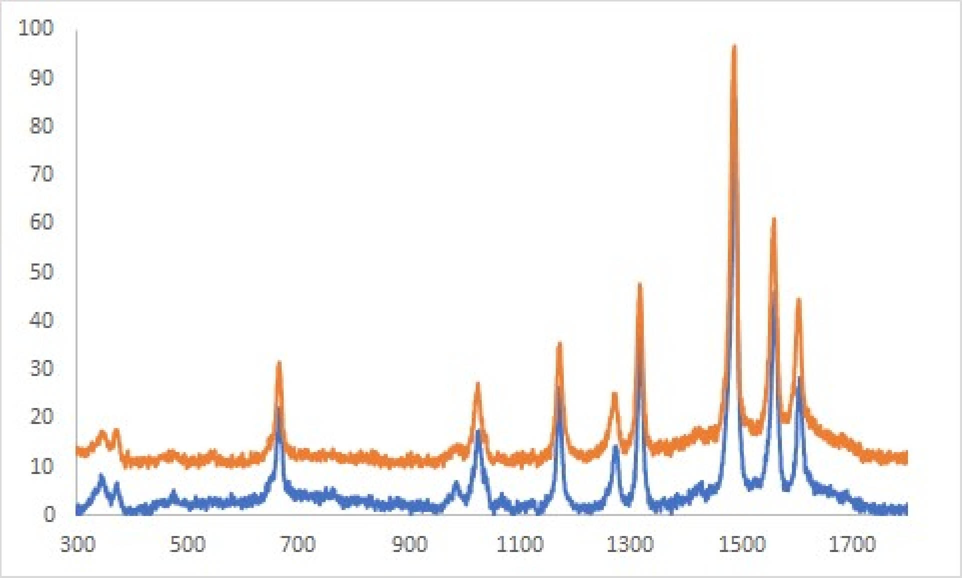

Figure S6. C. Resonance Raman spectra of 10^{-3} M **6** in water at pH 7 (blue) and of **6** with host **1** (10^{-3} M each)(orange).

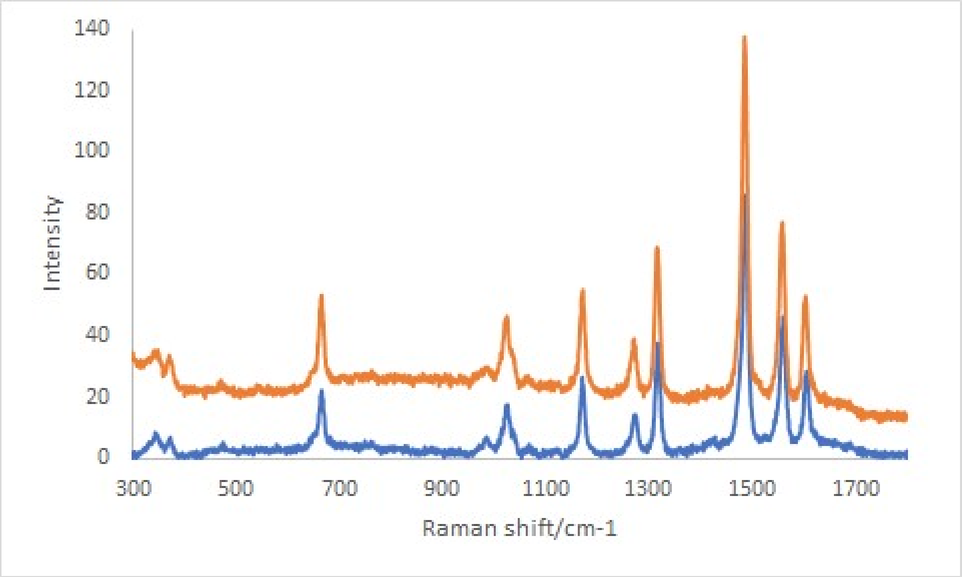

Figure S6. D. Resonance Raman spectra of 10^{-3} M **6** in water at pH 7 (blue) and of **6** with host **2** (10^{-3} M each)(orange).

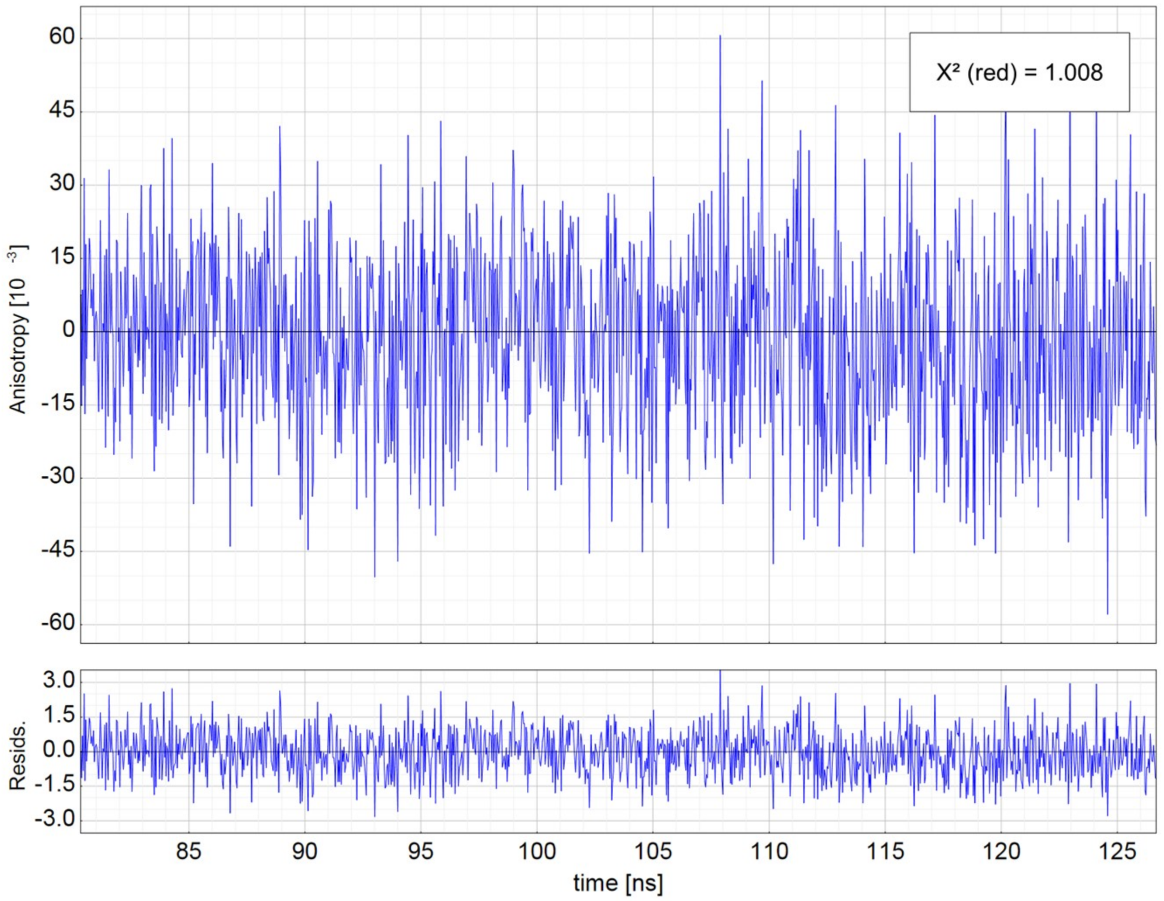

Figure S7. A. Time resolved emission anisotropy of 5x10^{-5} M **6** at pH 12. Excitation wavelength is 450 nm. Attempts to fit the data show no anisotropy. The exciting pulse is applied at 80.39 ns.

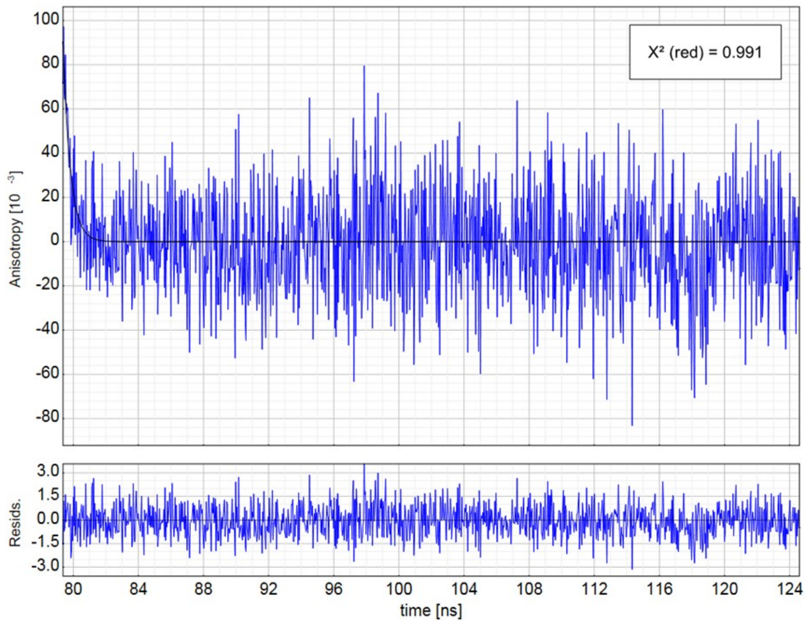

Figure S7. B. Time resolved emission anisotropy of 5x10^{-5} M **6** bound to 10^{-3} M host **1** at pH 12. Excitation wavelength is 450 nm and solid line shows fit to anisotropy decay that yields an R_0 of 0.09 and decay time of 0.3 ns. The latter value is beyond the resolution of our instrument which is approximately 1 ns. The exciting pulse is applied at 79.33 ns.

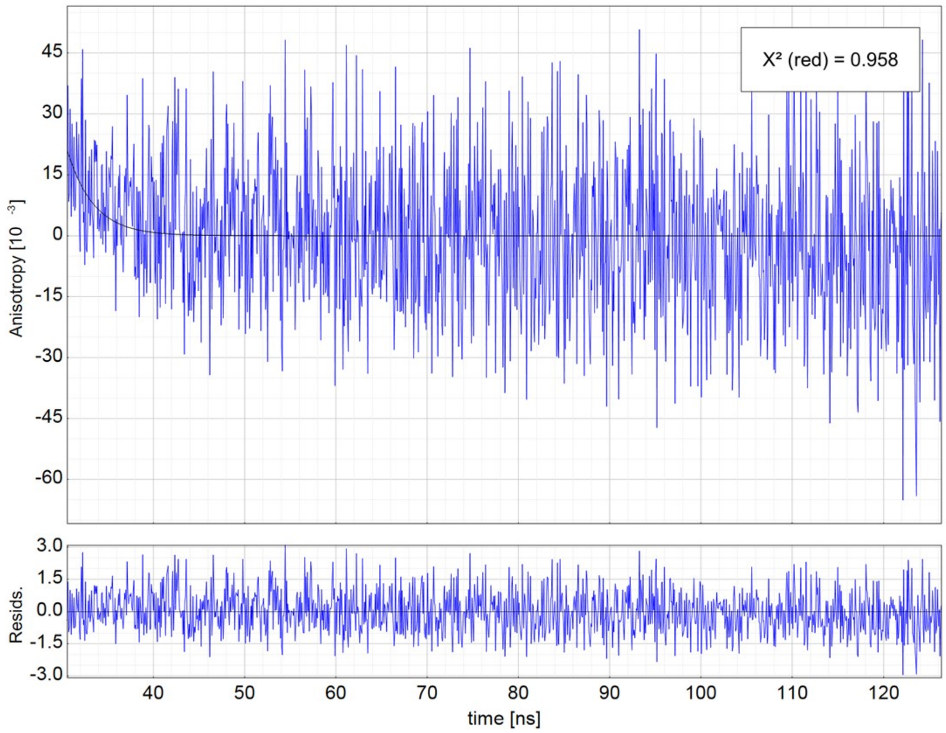

Figure S7. C. Time resolved emission anisotropy of 5x10^{-5} M **6** bound to 10^{-3} M host **1** at pH 7. Excitation wavelength is 450 nm and solid line shows fit to anisotropy decay that yields an R_0 of 0.028 and decay time of <3 ns. The exciting pulse is applied at 30.67 ns.

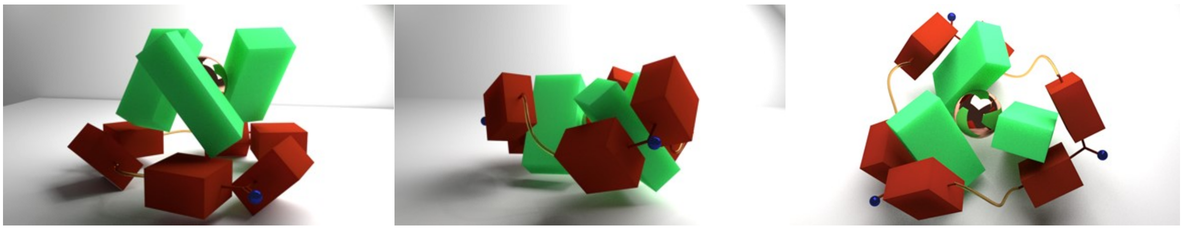

Figure S8. Schematic representations of host-guest complexes where all three ligands around Ru(II) are assumed to be bipyridine and where the hosts' carboxylate groups are omitted for clarity. Left: side view of **1·6** with perching binding. Middle: side view of **2·6** with inclusive/nesting binding. Right: top view of **2·6** with inclusive/nesting binding (gold ball = Ru(II), green box = bipyridine, red-brown box = phenylene, blue ball = corner oxygen, brown-yellow string = alkyl chain).
